# A pilot randomised controlled trial of the Peer Tree digital intervention targeting loneliness in young people: a study protocol

**DOI:** 10.1186/s13063-022-07029-7

**Published:** 2023-02-02

**Authors:** Michelle H. Lim, Lily Thurston, Robert Eres, Thomas L. Rodebaugh, Mario Alvarez-Jimenez, David L. Penn, Vassilis Kostakos, John F. M. Gleeson

**Affiliations:** 1 Prevention Research Collaboration, School of Public Health, University of Sydney, Camperdown, Australia; 2 Iverson Health Innovation Research Institute, Swinburne University of Technology, Hawthorn, Victoria Australia; 3 Orygen, The National Centre of Excellence in Youth Mental Health, Parkville, Victoria Australia; 4 Department of Psychology, RMIT University, Melbourne, Australia; 5 Department of Paediatrics, The University of Melbourne, Melbourne, Australia; 6 Neurodisability and Rehabilitation, Murdoch Children’s Research Institute, Melbourne, Australia; 7 School of Arts and Sciences, Washington University in St. Louis, St. Louis, USA; 8 Department of Psychology and Neuroscience, University of North Carolina, Chapel Hill, USA; 9 School of Behavioural and Health Sciences, Australian Catholic University, Melbourne, Victoria Australia; 10 School of Computing and Information Systems, University of Melbourne, Parkville, Australia

**Keywords:** Digital technology, E-mental health, Randomised controlled trial, Mental health, Strength-based, Loneliness, University students, Young adults

## Abstract

**Background:**

Young people are vulnerable to experiencing problematic levels of loneliness which can lead to poor mental health outcomes. Loneliness is a malleable treatment target and preliminary evidence has shown that it can be addressed with digital platforms. Peer Tree is a strength-based digital smartphone application aimed at reducing loneliness. The study aim is to reduce loneliness and assess the acceptability, usability, and feasibility of Peer Tree in young people enrolled at university.

**Methods:**

This will be a pilot randomised controlled trial (RCT) comparing a strength-based digital smartphone application (Peer Tree) with a control condition. Forty-two young people enrolled at university will be recruited for this pilot RCT. Participants with suicidal ideation or behaviours, acute psychiatric symptoms in the past month, or a current diagnosis of a mood or social anxiety disorder will be excluded. Allocation will be made on a 1:1 ratio and will occur after the initial baseline assessment. Assessments are completed at baseline, at post-intervention, and at follow-up. Participants in the control condition complete the same three assessment sessions. The primary outcome of the study will be loneliness. Depression, social anxiety, quality of life, acceptability, usability, feasibility, and safety of Peer Tree will also be measured as secondary outcomes.

**Discussion:**

This trial will report the findings of implementing Peer Tree, a smartphone application aimed at reducing loneliness in university students. Findings from this trial will highlight the initial efficacy, acceptability, and feasibility of using digital positive psychology interventions to reduce subthreshold mental health concerns. Findings from this trial will also describe the safety of Peer Tree as a digital tool. Results will contribute evidence for positive psychology interventions to address mental ill-health.

**Trial registration:**

Australian New Zealand Clinical Trial Registry ACTRN12619000350123. Registered on 6 March 2020

**Supplementary Information:**

The online version contains supplementary material available at 10.1186/s13063-022-07029-7.

## Administrative information

Note: the numbers in curly brackets in this protocol refer to SPIRIT checklist item numbers. The order of the items has been modified to group similar items (see http://www.equator-network.org/reporting-guidelines/spirit-2013-statement-defining-standard-protocol-items-for-clinical-trials/).

**Table:** 

Title {1}	A pilot randomised controlled trial of the Peer Tree digital intervention targeting loneliness in young people: a study protocol
Trial registration {2a and 2b}	Australian New Zealand Clinical Trial Registry, ACTRN12619000350123 Registered 6^th^ March 2020
Protocol version {3}	1.0 18 December 2020
Funding {4}	Swinburne University of Technology, Higher Education Participation and Partnerships Program (HEPPP) awarded to MHL.
Author details {5a}	Dr Michelle H Lim – Prevention Research Collaboration, School of Public Health, The University of Sydney & Iverson Health Innovation Research Institute, Swinburne University of Technology, Australia. Email: michelle.h.lim@sydney.edu.au Ms Lily Thurston – Iverson Health Innovation Research Institute, Swinburne University of Technology, Australia. Email: thurstonlily@gmail.com Dr Robert Eres – Department of Psychology, RMIT University & Iverson Health Innovation Research Institute, Swinburne University of Technology, Australia. Email: robert.eres@rmit.edu.au Prof Thomas L Rodebaugh – School of Arts and Sciences, Washington University in St. Louis, United States of America, Email: rodebaugh@wustl.edu Prof David L Penn – Department of Psychology and Neuroscience, University of North Carolina, Chapel Hill, United States of America, School of Behavioural and Health Sciences, Australian Catholic University, Melbourne, Australia, Email: dpenn@ad.unc.edu Prof Mario Alvarez-Jimenez – Centre for Youth Mental Health, The University of Melbourne, Orygen, Parkville, Australia, Email: mario.alvarez@orygen.org.au Prof Vassilis Kostakos – School of Computing and Information Systems, University of Melbourne, Parkville, Australia, Email: vassilis.kostakos@unimelb.edu.au Prof John FM Gleeson – School of Behavioural and Health Sciences, Australian Catholic University, Melbourne, Australia, Email: john.gleeson@acu.edu.au
Name and contact information for the trial sponsor {5b}	Swinburne University of Technology, PO Box 218, Hawthorn, Victoria, 3122
Role of sponsor {5c}	Conceptualisation, recruitment, design, and implementation of trial

## Background and rationale {6a}

Young people are vulnerable to experiencing high levels of loneliness. In Australia, 1 in 4 young people aged 18 to 25 years report problematic levels of loneliness [1, 2]. This is counterintuitive to common views of young people, given that young people are thought to be less socially isolated, often participating in school or work, and are competent users of digital technology [1]. While loneliness and social isolation are related, they are distinct concepts [3]. Loneliness is defined as a *subjective* experience of social isolation where there is a discrepancy between an individual’s desired and actual social relationships [4]. Loneliness may be more related to the quality of social relationships than to the quantity [5], and this may be more problematic for young people who may favour having a greater number of relationships over quality relationships [6].

Young adulthood is also known to be a period of transition and change. It is common for young adults to move out of home, separate themselves from established social networks and support systems, and be exposed to new social environments [7]. This is particularly true for young people at university; one key challenge in making a transition and adjusting to university is the formation of new peer groups [8]. Strong social bonds and reciprocal relationships with others have been found to be the strongest protective factor against loneliness in this population [9]. This is important as loneliness is an established risk factor for the development of mental ill-health in young people [1]. Therefore, addressing loneliness is a necessary step in improving the mental well-being of young people at university.

Young people have also been found to be particularly vulnerable to mental ill-health, with approximately 26% of those aged 16 to 24 experiencing mental health issues in Australia [10]. University students, compared with their non-university peers, report a higher prevalence of psychological distress with 19.2% of university students having very high levels of psychological distress (K10 score over 30), compared with 3% in the general population of the same age [11]. Higher levels of loneliness have been found to predict greater anxiety, stress, and depression in university students and increase the likelihood of dropping out from university [3, 12]. Thus, loneliness is an antecedent to poor mental health.

A key protective factor that reduces this risk is social connectedness and forming reciprocal bonds with peers [13–15]. While universities offer mental health and psychological support, young people are less likely to seek professional help [16], rather they rely on their peers for ‘informal’ mental health support [17, 18]. One way that young people rely on their peers is through digital technology. Therefore, the use of online mental health programmes could assist with engagement and compliance to treatments in this age bracket [17]. Previous research utilising online interventions to reduce various mental health symptoms has found positive results among both clinical (e.g. first-episode psychosis) and non-clinical samples of young people [19–22].

Fortunately, loneliness is feasible to address with online programmes [23–25]. In a recent trial, an 8-week Internet-based CBT intervention was administered to a community sample [26, 27]. Loneliness decreased significantly at the completion of the intervention as well as at the 2-year follow-up [26, 27]. Besides CBT, the majority of interventions targeting loneliness have used methods to improve social skills, enhance social support, and increase social contact [24]. Other digital interventions that hold the potential to address loneliness include those adopting strength-based, positive psychology interventions (PPIs). PPIs are designed to promote positive cognitions, feelings, and behaviours and improve overall psychological well-being [28]. More conventional psychological interventions target the amelioration of specific deficits (e.g. symptom reduction), but PPIs are designed to increase the sense of meaning and purpose in life and connectedness with others and enhance positive emotions [29–31]. A PPI targeting loneliness will build on participants’ social strengths and skills and will encourage the development of positive emotions within existing relationships by supporting users to show more prosocial behaviours towards others.

There is initial evidence in support of the efficacy of digital interventions for targeting loneliness in young people. In our previous pilot studies, we designed and developed +Connect, a PPI in the form of a smartphone application [32, 33]. Our pilot data supported the acceptability, feasibility, and safety of PPIs, as well as the potential for PPIs to reduce loneliness and mental health symptoms (i.e. social anxiety) in young people including with and without mental disorders [32, 33]. In a recent pilot RCT study, a smartphone application, Nod, employing a combination of positive psychology and cognitive and behavioural approaches, was used to address loneliness in university students [25]. Participants completed social challenges (e.g. acts of kindness, gratitude exercises) that were accompanied with written testimonials from young people over a 4-week period. Participants were also encouraged to provide reflections about their experiences in an attempt to restructure negative thoughts about social interactions. While initial data showed there were no significant effects in those reporting low depressive symptoms at baseline, Nod was found to be a safe and acceptable smartphone application that buffered the negative effects of loneliness in those who reported high levels of depression at baseline.

Recent evidence supports the need for interventions that target loneliness as a primary outcome rather than secondary to mental health [23]. Given that loneliness predicts subsequent deterioration in mental health outcomes, and current CBT models target mental health symptoms, it is important to address loneliness as a primary outcome. The study aim is therefore to reduce loneliness and improve mental health outcomes in vulnerable university students.

## Objectives {7}

The objective of this study is to evaluate the acceptability, feasibility, safety, and initial efficacy of a positive psychology smartphone application intervention called Peer Tree. Peer Tree is a digital smartphone intervention targeting loneliness and is a new iteration of our previous application +Connect. Peer Tree includes many features from +Connect [30, 31] but also includes the personalisation of modules based on user symptom profiles, a peer- and clinician-moderated chat forum, and animated videos to address loneliness in young people using a positive psychology framework.

It is hypothesised that Peer Tree participants, compared with the control participants, will report significantly lower loneliness at post-intervention, and these effects will remain at follow-up. It is also anticipated that Peer Tree participants, compared with control participants, will report significantly lower social anxiety and depressive symptoms, and higher quality of life and well-being outcomes at post-intervention as well as at follow-up.

## Methods

### Trial design {8}

**Fig. 1:**
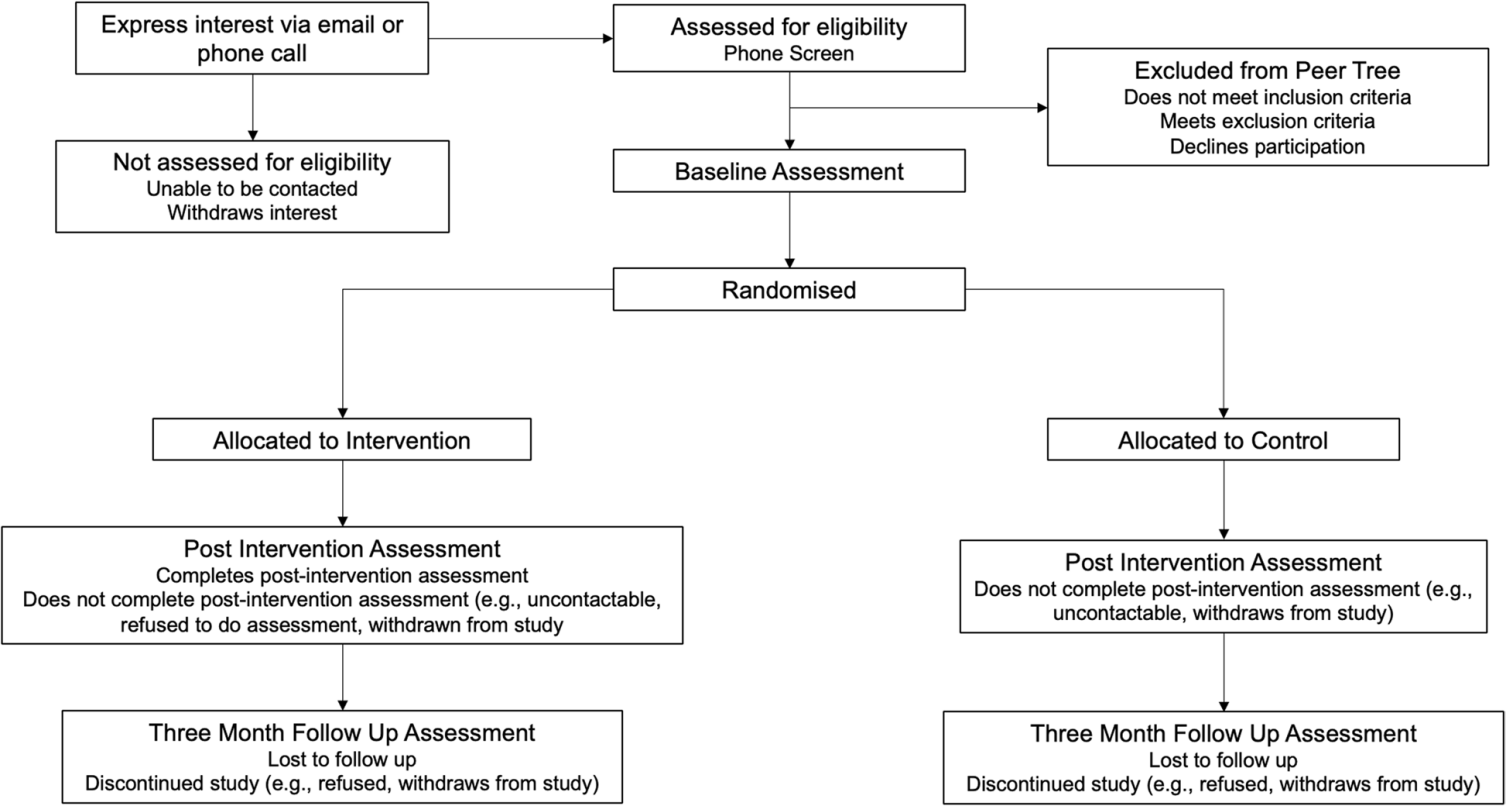
CONSORT flow diagram for the Peer Tree randomised controlled trial

Peer Tree is a pilot randomised controlled trial utilising a parallel groups design where participants are randomly allocated to either the Peer Tree intervention or control (i.e. No Peer Tree) group. There will be no blinding in this trial for assessors. Once determined to be eligible during the baseline assessment, participants will be randomised to one of the two conditions. Participants in both groups will complete a post-intervention assessment 6 weeks after the baseline or once they have finished the intervention. Participants in the Peer Tree arm will be given a maximum of 8 weeks to complete the smartphone application, after which they will complete the post-intervention assessment. Participants will then complete a follow-up assessment 3 months after the post-intervention assessment. Figure 1 displays the Consolidated Standards of Reporting Trials (CONSORT) flow diagram of the study procedure. The protocol was designed in accordance with the Standard Protocol Items: Recommendations for Interventional Trials (SPIRIT) and Good Clinical Practice Guidelines.

### Study setting {9}

This trial will be conducted at Swinburne University of Technology. Assessment sessions will be conducted in person at Swinburne University or online using a secure video conference service.

### Eligibility criteria {10}

Participants meeting the following criteria will be eligible for the study: (1) enrolled in any programme at an Australian university, (2) aged between 18 and 25 years, (3) English is their primary language, and (4) own a smartphone. Exclusion criteria are as follows: (1) self-reports acute psychiatric symptoms in the past month where the participant’s mental state has been worse than is usual for them (e.g. debilitating mood and anxiety symptoms), (2) reports any suicidal ideation or behaviour, (3) reports risk of harm to others or risk of damage to objects or property, (4) reports psychiatric hospitalisation in the past month, and (5) reports current diagnosis of a mood disorder or social anxiety disorder.

### Who will take informed consent? {26a}

During the initial assessment, a researcher will obtain verbal and written consent from the participant.

### Additional consent provisions for collection and use of participant data and biological specimens {26b}

During the baseline assessment, participants will have the option to allow their data to be used in future studies. This data will be aggregated to a group level, and no identifiable information will be used in any future studies.

## Interventions

### Explanation for the choice of comparators {6b}

As our focus is the examine the effectiveness of the intervention above and beyond an individual’s usual activities, we selected a control group who did not receive the Peer Tree intervention and compared it to a group who received Peer Tree intervention.

### Intervention description {11a}

#### Features of the digital intervention

##### Modules

Peer Tree delivers a series of 42 psychoeducational modules in the form of videos, animations, and images over a 6–8-week period. Each module helps develop core skills for a particular topic. Peer Tree delivers a series of 42 psychoeducational modules in the form of videos, animations, and images over a 6–8-week period. Each module helps develop core skills for a particular topic. There are three types of videos that participants will watch: animation (narrated animation videos illustrating concepts), actor (actors role-playing concepts), and shared experience (young people with lived experience of loneliness discussing concepts). Each module takes approximately 2–6 min to complete, with new modules being released each day to the user only if they had completed the previous module.

##### Challenges

Participants will also be asked to participate in challenges. These are designed to help apply what participants learn within the application in real life. If participants choose to take on a challenge, they will have between 1 and 7 days to complete the challenge depending on the module. Once participants complete a challenge, for example, doing 5 acts of kindness per day, they will earn a token.

##### Tokens

As participants progress through Peer Tree, they will receive tokens on the home screen. Participants will earn tokens depending on their interaction with the application. For instance, if participants have posted a certain number of posts in the chat forum, they will earn a token.

##### Chat forum

Users of Peer Tree also have access to a moderated chat forum where they can discuss each module with their peers. A one-on-one chat function is also available for participants to contact other participants or moderators privately. There are four types of moderation in the peer tree study, all of which are in place to ensure participant safety. These include clinical, peer, research, and technical moderation.

##### Moderation manual and training

A training manual has been developed detailing the responsibilities and duties of each kind of moderator (detailed below) as well as the protocol for managing serious adverse events and risk issues. This manual will be used as a reference for all moderators and the research team throughout the trial. The research team and peer moderator will undergo training in administering the Structured Clinical interview for Diagnostic and Statistical Manual for Mental Disorders (DSM-5)–research version (SCID-5-RV) and assessing and managing risk and this will be conducted by the principal investigator (PI) who is a registered clinical psychologist. The PI will only permit someone to be part of the research or moderation team once they received training on identifying and managing risk and demonstrate competence on following the risk protocol procedures.

##### Clinical moderation

A mental health professional with experience in recognising potentially dangerous or risky behaviour will monitor the chat forum. If a clinical risk (e.g. intention to harm self or others) is detected, the clinical moderator will follow up directly with the young person and aid as necessary. Clinical moderators will monitor the forums every day. See Supplementary material for the standardised operating procedure on reporting identifiable risks.

##### Peer moderation

A peer with similar demographics to the participants will be hired to act as a peer moderator on the chat forum. The role of the peer moderator will be to engage the participants and facilitate discussion on the forums. If the peer moderator identifies any potentially dangerous or risky behaviour, they will notify the clinical moderator directly. The peer moderator will be active every day with the exception of weekends.

##### Research moderation

The research team will moderate the forums daily to ensure the intervention is running smoothly and to assist with any inquiries. Research moderators will also engage with participants in the forums. If any research moderators identify any potentially dangerous or risky behaviour, they will notify the clinical moderator directly. Research moderators will be active every day.

##### Technical moderation

There is an automated keyword function embedded within the smartphone application that is activated each time a participant posts a contribution containing potentially offensive words. Any participant who tries to use one of these words or phrases will be blocked from doing so and a notification will be sent to the research team. The research team or clinical moderator will assess the notification and follow up on the issue if necessary.

##### Weekly check-ins

Each week, a member of the research team will call the participant for a short conversation about their progress, any technical issues experienced, and to answer any of their questions.

### Criteria for discontinuing or modifying allocated interventions {11b}

Participants are free to withdraw at any time without prejudice or discrimination. Participants who discontinue from the project will always be asked their reasons behind their discontinuation from the study. They are not obligated to disclose these reasons.

If an adverse event (i.e. a participant displays risk to self, others, or objects) is identified, this will be followed up until a resolution has been made, or the participant is stabilised. Throughout the course of the intervention, the research team and moderators on the application will monitor for deterioration in participants’ mental states. If a deterioration is detected, a risk assessment will be conducted. In the event that the participant is deemed a risk to themselves or others, they will be removed from the intervention and referred on to get professional support.

Participants may also be considered withdrawn from the study if the research team is unable to contact the participant on three consecutive attempts. If the research team is successful in contacting a participant, the issue will be escalated to the PI or a clinical moderator to continue follow-up. If the researcher cannot contact a participant where there are concerns for well-being, their emergency contact will be contacted. Participant data collected up to the time of the withdrawal will be included in data analysis if it makes sense to do so.

### Strategies to improve adherence to interventions {11c}

A weekly phone check-in will be completed by a member of the research team for the duration of the intervention. During this check-in, the researcher will answer any questions the participants have and encourage them to engage with the content and chat forum if they are not currently.

### Relevant concomitant care permitted or prohibited during the trial {11d}

All participants will be able to continue or commence any mental health or psychological care as required.

### Provisions for post-trial care {30}

This study does not deal with a clinical population. All participants are screened using a semi-structured clinical interview to ensure they do not meet the criteria for a psychiatric illness. Thus, any participant accepted into the programme is unlikely to require post-trial care. Once the trial is completed, there is no further follow-up. The exception to this is if a participant is withdrawn from the study due to a deterioration in mental health. In this case, we will follow up with the participant to ensure they are receiving adequate support.

### Outcomes {12}

See Table 1 for the SPIRIT schedule of measures used in this study. The primary outcomes are loneliness, acceptability, feasibility, and safety of Peer Tree. Secondary outcomes include social anxiety, depression, quality of life, and psychological well-being. Demographic variables will include age, gender, sexuality, ethnicity, work status, level of education, previously attempted university degrees, current university course load, postcode, and religion.

**Table 1:** SPIRIT schedule of outcome measures

Outcome measures	Baseline	End of treatment	Follow-up
Enrolment			
Eligibility screen	X		
Informed consent	X		
Allocation	X		
Assessments			
Demographic form	X		
UCLA-LS3	X	X	X
SIAS	X	X	X
CES-D	X	X	X
LSNS-18	X	X	X
IIP-32	X	X	X
PWB-54	X	X	X
PANAS-SF	X	X	X
WHO QOL-Bréf	X		
COVID-19 question	X	X	X
Adherence		X	
Mean Peer Tree application acceptability		X	
Mean Peer Tree application feasibility		X	
Mean Peer Tree application usability		X	

*UCLA-LS3* UCLA Loneliness Scale-Version 3, *SIAS* Social Interaction Anxiety Index, *CES-D* Centre for Epidemiological Studies-Depression, *LSNS* Lubben Social Network Scale, *IIP-32*, Inventory of Interpersonal Problems, *PWB* Scales of Psychological Wellbeing, *PANAS-SF* Positive and Negative Affect Scale–Short Form, *WHO QOL-Bréf* World Health Organization Quality of Life-Bréf

#### Primary outcome

##### UCLA Loneliness Scale-Version 3 (UCLA-LS; 36)

The UCLA-LS is a 20-item measure employing a 1 (never) to 4 (always) ordinal scale [34]. The measure consists of both positively and negatively worded items that assess loneliness (e.g. How often do you feel that you are no longer close to anyone?). The UCLA-LS has been shown to correlate negatively with life satisfaction and perceived social support, thus supporting its convergent validity with related constructs [35].

#### Secondary outcomes

##### Centre for Epidemiological Studies-Depression (CES-D; 38)

The CES-D is a 20-item self-report measure of depressive symptoms, which employs a 0 (rare or none of the time) to 3 (most or all of the time) Likert-type scale. Scores are summed to create a total score indicative of depression symptomatology, where higher scores indicate the presence of more symptomatology. The CES-D has strong internal reliability [36]. The CES-D has strong internal consistency ranging from *α* = .88 to .90 across time [37].

##### Social Interaction Anxiety Index (SIAS; 39)

The SIAS is a 20-item measure employing a 0 (not at all characteristic of me) to 4 (extremely characteristic of me) Likert-type scale. The 17 straightforward items (i.e. items that are not negatively worded) will be used here as they have previously been deemed more reliable than the full 20-item measure [38]. The measure involves statements describing one’s thoughts, feelings, and behaviours to social situations. The SIAS has demonstrated excellent internal consistency (*α* = .88 to .93; *α* = .88 to .93) [38, 39].

##### Lubben Social Network Scale (LSNS; 41)

The LSNS is an 18-item scale that assesses the frequency and quality of social contact—such as talking about private matters—in an individual’s network. There are three subscales, each consisting of 6 items relating to family, neighbour, and friend social ties. The scale employs a 0 (none) to 5 (nine or more) Likert scale and includes 6 items (e.g. How many relatives do you see or hear from at least once a month?) which are summed together to provide a total score. Higher scores indicate larger social networks and lower risk of social isolation. The scale has demonstrated adequate levels of internal consistency (*α* = .93 for the total LSNS) and the proposed clinical cut-points showed good convergent validity [40].

##### The Scales of Psychological Well-Being (SPWB; 42)

The SPWB is a 54-item questionnaire that measures psychological well-being across six dimensions: autonomy, positive relations with others, environmental mastery, personal growth, purpose in life, and self-acceptance. Items are scored on a 6-point Likert scale from 1 (strongly disagree) to 6 (strongly agree). This widely used scale has demonstrated good internal consistency and construct validity [41]. Internal consistency for the SPWB ranged from *α* = .81 to .87. We will use an overall SPWB score by adding the scores for each subscale.

##### The Positive and Negative Affect Schedule (PANAS-10; 43)

The PANAS-10 is a 10-item subscale measure of the PANAS scale. The measure asks respondents to rate the extent to which they feel a particular emotion along a 5-point Likert scale ranging from 1 (very slightly) to 5 (extremely). This subscale measure has been shown to have good internal consistency (*α* = .88) [42]; and acceptable levels of convergent validity [43].

##### World Health Organisation Quality of Life (WHO QOL-BRÉF; [44])

The WHO QOL-BRÉF is a 26-item abbreviated version of the WHO QOL-100, a quality-of-life assessment which is cross-culturally valid. The WHO QOL-BRÉF contains one item from each of the 24 facets of quality of life in the WHO QOL-100, plus two additional items on the overall quality of life and general health. The WHO QOL-BRÉF has four domains of quality of life: physical health, psychological, social relationships, and environment. The domains physical health, psychological, and environment have good internal consistency (*α* = .82, .81, .80 respectively) and the social relationship domain has acceptable internal consistency (*α* = .68) [45].

##### Peer tree acceptability

Acceptability will be assessed in the same way as our previous pilot trials see [32, 33]. Acceptability will be assessed post-intervention using satisfaction ratings on application enjoyment and usefulness and content helpfulness. A set of 22 satisfaction ratings will be measured using a 5-point Likert scale from 1 (*extremely disagree*) to 5 (*extremely agree*), with an example item being ‘I enjoyed using the app’. A further 15 satisfaction ratings were adapted from Meyer et al. (2012) and involve participants making ratings on a 3-point Likert scale from 0 (*not at all satisfied*) to 2 (*very much so*), an example item being ‘Did the intervention help you to feel more connected with others?’ [31]. Finally, participants will be asked to rate how helpful they found each Peer Tree module (e.g. gratitude) on a 4-point Likert scale from 1 (*not at all helpful*) to 4 (*very helpful*).

##### Peer tree feasibility

Feasibility will be assessed by considering four key factors: uptake, attrition, retention, and application completion. Uptake is defined as the number of potentially eligible young people who attended the baseline assessment. We set an apriori threshold of at least 50% of people who were eligible would attend a baseline assessment. Attrition is defined as the number of participants who attended the baseline assessment but failed to log into the app for more than three consecutive days and where researchers were unable to contact the participant. We set a priori threshold of a 30% attrition rate. Retention is defined as the number of participants who complete the intervention. Application completion is defined as accessing and completing at least 50% of the app (21 out of 42 days) within the maximum allocated time of 8 weeks. These criteria are consistent with our previous pilot trials of +Connect [32, 33].

##### Peer tree safety

Safety is operationalised as the incidence of serious adverse events during the course of the study as a result of the Peer Tree intervention. Serious adverse events are defined in this trial as harm to self (including non-suicidal self-injury and suicide), risk of harm to others (including homicidal risk), and risk of harm to objects or property. Adverse events will be actively assessed and monitored throughout the intervention and managed accordingly as described in the risk assessment protocol located within the Supplementary files. The intervention will be deemed safe if there are no reported adverse events related to the trial.

#### Covariates

##### Inventory of Interpersonal Problems (IIP-32; 47)

The IIP-32 measures difficulties people experience in their interpersonal relationships. For example, things people find ‘too hard’ or do ‘too much’. The IIP-32 taps into 8 domains which includes hard to be assertive, hard to be sociable, hard to be supportive, too dependent, too caring, too aggressive, hard to be involved, and too open. Respondents answer each item using a scale ranging from 0 (not at all) to 4 (extremely). Internal consistencies for each domain have been shown to be acceptable: assertive: *α* = .86, sociable: *α* = .89, supportive: *α* = .75, dependent: *α* = .71, caring: *α* = .72, aggressive: *α* = .85, involved: *α* = .75, and open: *α* = .80. Overall, the internal consistency for the IIP-32 is good .86 [46].

##### COVID-19 impact

The impact of COVID-19 on the participant in the prior 7 days will be assessed using a single-item measure. The item is measured on a 5-point Likert scale from 1 (not at all affected) to 5 (greatly affected).

#### Exploratory

##### Usability

Consistent with previous trials of +Connect, usability will be assessed post-intervention using an 8-item satisfaction rating scale. Each item ranges from 1 (extremely disagree) to 5 (extremely agree). Items relate to the format, font, colour scheme, and how easy the application was to navigate and use.

### Participant timeline {13}

The participant timeline is as follows:

- Screening: a brief phone call or face-to-face assessment to verify initial inclusion and exclusion criteria.
- The baseline assessment and secondary verification procedure will be conducted 1–7 days after screening.
- If eligible, participants will be randomised to either Peer Tree or control.
- Peer Tree participants will wait until a suitable number of participants is available to be onboarded onto the Peer Tree application. This will be dictated by recruitment speed. Once enough participants have been randomised to Peer Tree, the intervention will begin. After 6–8 weeks, participants will be seen for the post-intervention assessment.
- Control participants will wait for 6 weeks before starting the post-intervention assessment.
- Three months following the completion of the post-intervention assessment, participants will complete the third and final assessment. Once the follow-up assessment is complete, the study is completed.

### Sample size {14}

A power analysis was conducted using G*Power 2.0 software. The *F* statistic function for an intervention design was used to obtain the relevant sample size. This is established using loneliness outcome data and is estimated using the effects sizes derived from +Connect. Loneliness was used to determine statistical power as it was the target variable of our primary outcomes. This revealed that 42 participants are needed to detect a moderate effect with 80% power.

### Recruitment {15}

Recruitment will take place from three main channels: (1) flyers posted around university campuses, (2) social media posts with links to the study will be posted on university-affiliated pages and through paid Facebook and YouTube adverts, and (3) through emails to list of students who access different services at the sponsor university.

Students who express interest in the study will be contacted by a member of the research team for an initial phone screen to provide more detailed information about the study and assess eligibility for the baseline assessment. The phone screen comprises of a standardised set of questions that assesses the interested student’s suicide ideation or behaviour risk, risk of harm to others and property, and mental health status (i.e. the presence of acute psychiatric symptoms in the past month) to ensure they are suitable for the baseline assessment. The phone screen will not guarantee eligibility and potential participants are informed of this at the outset. If the participant is still interested and deemed eligible at the end of the phone screen, a time is scheduled for the baseline assessment, during which eligibility is confirmed through a more comprehensive assessment.

Participants are reimbursed with a $50 e-Gift card for their time attending each of the three assessment sessions. If during the baseline assessment a student is found to be ineligible, they are reimbursed for their time with a $15 e-Gift card.

## Assignment of interventions: allocation

### Sequence generation {16a}

The baseline assessment session consists of two parts. The first confirms eligibility through an assessment of the mood and social anxiety modules in the Structured Clinical Interview for DSM-5 Research (SCID-5-RV). If a participant is found to be ineligible due to a SCID-5-RV diagnosis or the presence of risk issues, they will be referred to the Swinburne Psychology Clinic and a national crisis service (e.g. Lifeline). If a participant is eligible at the completion of the SCID-5-RV, they will be subject to random allocation using a computer-generated sequence. Randomisation to condition will be made based on Kim and Shin’s (2014) recommendation to use randomisation.com to allocate participants based on blocks to one of either Peer Tree or control.

### Concealment mechanism {16b}

There will be no concealed allocation in this trial due to the nature of the intervention. Participants will either be given Peer Tree or not and therefore explicitly know which condition they are in. The purpose of Peer Tree is to teach positive psychology strategies in an easy-to-use format and augmented with a safe, virtual space to practice and reflect on the strategies learnt. Additionally, concealment will not be viable as part of the treatment arm includes the research team establishing a close relationship with the participants in the intervention to ensure a safe environment and to help guide participants through Peer Tree to maximise the positive effects of the intervention.

### Implementation {16c}

The sequence allocation will be generated by an independent researcher who will provide the participant’s allocation to the team member conducting the baseline assessment. This document will be stored securely on Swinburne University of Technology’s online servers and only investigators who are engaged in the project will have access to this. The document will also be password protected. The allocation will be provided to the assessor while they are in the baseline assessment via text message.

## Assignment of interventions: blinding

### Who will be blinded {17a}

Due to the nature of the trial, no participants or researchers will be blinded to condition. Assessors will be blind to experimental condition until the end of the baseline assessment. They will request the condition from an independent researcher who will confirm the allocation to the experimental condition. Research members who conduct the assessments will also be a moderator and will help to onboard participants onto the Peer Tree platform. We opted for no blinding to allow participants to have a familiar person they can engage with on the Peer Tree platform if needed.

### Procedure for unblinding if needed {17b}

Not applicable.

## Data collection and management

### Plans for assessment and collection of outcomes {18a} and data management {19}

Data will be collected in the following formats: paper, electronic survey database, and digital audio. All data will be coded with a unique numerical identifier. A list of names of the recruited participants and their corresponding numerical identifiers will be kept in a password-protected digital spreadsheet only accessible to project personnel named in this protocol. Electronic survey data will be kept in a Qualtrics account only accessible by the research team. No identifiable information will be collected in Qualtrics and responses will only be identified by a unique numerical identifier.

Other non-electronic identifying details (e.g. consent forms) will not be stored with research data. All paper notes will be kept for at least 5 years post any publication of results in a locked filing cabinet. Data will be shredded and disposed of securely after this time. Digital audio files will not contain any identifiable information about participants, for example, they will not be asked their name during the audio-recorded interview. Digital audio files will be labelled with participants’ unique numerical identifier and stored on a USB drive which will be locked in a filing cabinet. The password is available only to the research team. All files are protected using 128-bit encryption methods which are the gold standard in document protection. Passive data from the Peer Tree will also be collected. This information is collected and stored on secure online servers in line with Australian standards. This is part of the application development and it will not collect any information that can personally identify the participant.

### Plans to promote participant retention and complete follow-up {18b}

Participants in the intervention will be contacted weekly for the duration of the trial to promote engagement and retention through the intervention. Participants in control will be contacted 2 weeks before each assessment to schedule the next assessment. A reminder will be sent via text message a day prior to the assessment.

### Confidentiality {27}

All data will be stored with a unique numerical identifier rather than the participant’s name. All identifiable documents will be stored in a locked filing cabinet separate from assessment documents.

### Plans for collection, laboratory evaluation, and storage of biological specimens for genetic or molecular analysis in this trial/future use {33}

Not applicable. No biological specimens are being collected.

## Statistical methods

### Statistical methods for primary and secondary outcomes {20a}

All data processing and analysis will occur either in the statistical package for the Social Sciences (SPSS) or Mplus. Data manipulation will only occur if the data violates the statistical assumptions for the tests that we would be conducting. In this instance, the data will be transformed into a format that does not jeopardise the interpretability of the scores.

An intention-to-treat (ITT) analysis will also be conducted on all participants who were enrolled in the project but dropped out of the study. A mixed-model analysis will be used to determine whether a greater change in loneliness occurs over time for the Peer Tree group compared with the control group. This form of analysis creates an unbiased view of the intervention by including every participant who was assigned to each group in the final analysis, rather than those who contributed to each data point in the study. That is, if 15 people were assigned to an intervention but only nine of them completed the intervention, the ITT analysis would include all 15 participants in the analysis to provide an appropriate measure of what the effect would look like with every participant included.

A per-protocol analysis using mixed-model analysis of variance will be conducted to determine whether a change in loneliness occurs over time more so for the Peer Tree group compared with the control condition. Time (i.e. baseline, post-intervention, follow-up) will be the repeated measure factor, while intervention (i.e. Peer Tree vs control) will be the between-participant factor. Two-tailed Pearson’s *r* correlations will be calculated to assess the relationships between our primary outcome, loneliness, and secondary outcomes. This will be used to determine the suitability of conducting additional, regression-based analyses. In the instances that there are violations to the assumptions of these tests, we envisage that any combination of the following will be used to answer our focal questions: standard and hierarchical regression, mediation and multiple mediation, latent trajectory modelling, *t*-tests, and mean comparisons.

### Interim analyses {21b}

No formal interim analyses are planned during the trial. The data safety committee may have preliminary views of the data to ensure safety.

### Methods for additional analyses (e.g. subgroup analyses) {20b}

There is potential for subgroup analyses to be conducted; however, these have not been planned or decided upon yet.

### Methods in analysis to handle protocol non-adherence and any statistical methods to handle missing data {20c}

Missing data from participants who have withdrawn from the study will not be dealt with using data imputation; instead, intention-to-treat analyses will be used to provide an evaluation of the intervention. Missing data at the variable level will not be imputed if less than 5% of data is missing.

### Plans to give access to the full protocol, participant-level data, and statistical code {31c}

The datasets analysed during the current study trial, as well as the statistical code, are available from the corresponding author upon reasonable request. Any data that is made available will be at the group level and completely de-identified.

## Oversight and monitoring

### Composition of the coordinating centre and trial steering committee {5d}

The steering committee consists of the named authors on this protocol. All members are independent of the sponsor. The committee members have either contributed to the design of the trial and provide ongoing support or are active investigators in the trial or both.

### Composition of the data monitoring committee, its role, and reporting structure {21a}

A data safety monitoring committee made up of 3 additional representatives will ensure governance of these data including mitigating the risk of potential safety breaches. This committee is separate to the sponsor and the steering committee.

### Adverse event reporting and harms {22}

An adverse event within this study is defined as participants reporting more than discomfort (e.g. distress) over a particular aspect of the current study. Participants are screened for clinical risk of harm to self, others, and objects or property during the phone screen and during each of the assessments.

Due to the nature of the smartphone application content, we anticipate minimal risk to participants. Participants will not be coerced into answering questions or asked to contribute if they feel uncomfortable, and they can choose not to answer any questions in the packet of measures at the assessment sessions. Participants will be able to withdraw their participation in the smartphone application intervention at any time. We have included a contact section in the smartphone application where there will be information about referral options to a national crisis service.

In the unlikely event of any participant experiencing significant ongoing distress during their participation, the researchers will actively assist the participant in obtaining any additional support they may require. Our protocol for doing this is to provide contact numbers for a national crisis service and/or the University Counselling Service and prioritising the person accessing support from services in their local area that can be most responsive to their needs. The researchers will support the person in getting in touch with their desired health services (e.g. local GP) or offer to do so on their behalf. This will only be done with their permission. In the unlikely scenario that a participant is unwilling for services to be contacted and the person presents a risk to themselves or others, the researchers will have a duty of care to contact services without the person’s permission. This possibility is highlighted in the participant information sheet. The PI is responsible for evaluating all adverse events in the trial and reporting all relevant details to the local ethics committee and mental health service.

### Frequency and plans for auditing trial conduct {23}

Audits may be conducted by the research team to ensure trial conduct is adhered to. This will be separate to routine monitoring of trial conduct with the protocol, good clinical practice, and ethical guidelines.

### Plans for communicating important protocol amendments to relevant parties (e.g. trial participants, ethical committees) {25}

The PI will notify the sponsor and trial research team of any change or alterations to the protocol. All changes will be sent to review by the local research ethics committee. The PI will update the protocol on the clinical trial registry.

### Dissemination plans {31a}

Upon completion of the study, the findings from this study will be made publicly available through four main avenues. First, research findings will be reported and disseminated in peer-reviewed journal articles. This will be accessible to people through paid subscriptions and through tertiary institutions. Second, research findings will be presented at scientific conferences, and third, a summary of the findings will be made available on the Social Health and Wellbeing Laboratory website where participants can review this information. Finally, a summary of the findings may also be sent to participants if they so choose. In any case, the dissemination of these findings will be completely anonymous and only data collated into group means will be reported. No identifiable information will be published.

## Data safety management committee

An internal data safety management committee will ensure participation in the study is not a burden or harmful to the participants. The committee made up of three independent researchers will meet every 3 months and evaluate a de-identified dataset to ensure that no risk-related patterns are being presented. De-identification of the dataset will be completed by a research assistant on the project who will not be on the committee. The inclusion of the data safety management committee will allow for early detection of risks and problematic behaviours.

## Discussion

Students often find the transition from secondary school to university challenging [7, 8], making this population more vulnerable to feeling lonely. Despite the perception that young people are well connected, 1 in 4 young people experience problematic loneliness [1, 2]. Higher levels of loneliness are associated with worse mental health symptoms [3, 12] and difficulties adjusting to university and forming strong reciprocal bonds with peers [9].

The aim of the pilot randomised controlled trial is to determine the initial efficacy, acceptability, and safety of Peer Tree, a digital positive psychology intervention aimed at reducing loneliness in tertiary students. Results from this study would support the notion that digital interventions are a cost-effective tool to help mitigate loneliness in university students in order to facilitate better mental health and well-being. This study will provide evidence for the utility of digital positive psychology interventions for strengthening relationships in university students.

## Trial status

Two years from 2020 to 2021. Protocol version 1.0. Data collection has started and is ongoing and is expected to be completed by June 2021. Delayed submission due to the COVID-19 pandemic.

## Supplementary Information

**Additional file 1.**

## Data Availability

The datasets during the current study are available from the corresponding author on reasonable request.

## Abbreviations

RCT: Randomised controlled trial
CONSORT: Consolidated Standards of Reporting Trials
SPIRIT: Standard Protocol Items: Recommendations for Interventional Trials
UCLA-LS-3: University of California Los Angeles Loneliness Scale-Version 3
CES-D: Centre of Epidemiological Studies-Depression
SIAS: Social Interaction Anxiety Index
LSNS: Lubbens Social Network Scale
SPWB: Scales of Psychological Wellbeing
PANAS: Positive and Negative Affect Scales
WHO QOL-BRÉF: WHO Quality of Life-Bréf
IIP: Inventory of Interpersonal Problems
ITT: Intention to treat
SPSS: Statistical Package for Social Sciences
PI: Principal investigator
