# Supplementary material for: A pilot randomised controlled trial of the Peer Tree digital intervention targeting loneliness in young people: a study protocol
Source: Trials. 2023 Feb 2;24:77. doi: 10.1186/s13063-022-07029-7 (PMC9893200; doi:10.1186/s13063-022-07029-7)
Supplement: Supplementary file 1 — Additional file 1. [file 13063_2022_7029_MOESM1_ESM.docx]

Supplementary Material

Figure 1 Standardised operating procedure for identifiable risks observed during the Peer Tree program

Report to ethics

Continue monitoring this participant and remove inappropriate content

Emergency (000) or a crisis assessment and treatment team

**If any risk identified:**

**Low Risk**

Contact with participant

**Moderate Risk**

**High Risk**

  Stop participant’s Peer Tree usage and follow risk protocol

Stop participant’s Peer Tree usage and follow risk protocol

Research Moderator /

Principal Investigator

**After hours procedure**

**9am – 5pm Monday - Friday**

Clinical Moderator

Inform Research Moderator /

Principal Investigator

*Note: Peer moderators will report any adverse event/risk to either the clinical or the research moderator.

# Risk Assessment

A risk assessment will help determine the participant’s current level of risk and determine the most appropriate response. Below is a guide on the safety protocol for risk to self and others:

## i) Risk of harm to self

Individuals who are assessed reporting any level of risk for suicide will **not** be accepted into the program and will be encouraged to seek supportive health services e.g. calling Lifeline (13 11 14) or engaging with a mental health service such as the Swinburne Psychology Clinic (T: 9214 8653).

Mild risk of suicide is characterized by fleeting, vague thoughts of suicide with no plan to follow through on those thoughts. The individual demonstrates good judgement and insight and is able to verbalise their own safety plan and identify the protective facts they have in place.

*Protocol:* Individuals who are assessed as at *mild risk* for suicide will be encouraged to seek supportive health services, e.g. calling Lifeline (13 11 14), engage with the Swinburne Psychology Clinic or other external support services in their local area. If a participant is currently engaged with a mental health professional, the participant will be encouraged to contact their existing professional for support.

Moderate risk of suicide is characterised by more frequent thoughts of suicide with a vague plan of how they would follow through with their suicidal thoughts and a vague intent to die. The individual demonstrates fair judgment but has a safety plan in place.

High risk of suicide is characterised by intense frequent thoughts of suicide, a preoccupation with the thought of ending their life and a clear plan as to how they would follow through on those thoughts. The individual demonstrates poor judgement and does not have a safety plan in place.

*Protocol:* Individuals who experience a moderate to high risk of suicide will be encouraged to access supportive health services e.g. calling Lifeline (13 11 14) or engage with the Swinburne Psychology Clinic (T: 9214 8653) or other external support services in their local area, or their current mental health professional, if they are already engaged with a service. The participant’s emergency contact nominated in the baseline assessment will also be notified via phone call on the participant’s behalf about the reported risk to self and/or others. This will be communicated with the participant prior to the emergency contact being notified. In some cases of moderate to high risk, the participant may have their access to Peer Tree temporarily suspended.

Depending on the immediacy of the risk, research members may decide to access health and emergency services and/or an emergency contact on the individual’s behalf. This will only be done with their permission, unless in the highly unlikely scenario arises that the person is unwilling for services to be contacted and the person presents a high risk to themselves or others. In this circumstance, the research will have a duty of care to contact services without the person’s permission. This possibility is highlighted in the participant information sheet when the participant completes the baseline assessment.

The researchers are advised to seek supervision from a senior research member (Dr Michelle Lim) to discuss participant’s wellbeing, the nature of the risk, adherence to protocol and outcome of the situation.

## ii) Risk of harm to others

Individuals who report having moderate to high risk issues relating to thoughts of harming others will not be offered a place in Peer Tree trial.

Mild risk of harm to others is characterized by fleeting, vague thoughts of hurting others with no plan to follow through on those thoughts. The individual demonstrates good judgement and insight and is able to identify protective factors they have in place.

Individuals who are assessed as at mild risk for hurting others will be encouraged to seek supportive health services, e.g., calling Lifeline (13 11 14), engage with their case manager and/or mental health treating team or other external support services in their local area. If a participant is currently engaged with a mental health professional, the participant will be encouraged to contact their existing professional for support.

Moderate risk of harm to others is characterized by more frequent thoughts of harming others with a vague plan of how they would follow through with their thoughts and a vague intend to cause harm. The individual demonstrates fair judgment but has a safety plan in place.

High risk of harm to others is characterized by intense frequent thoughts of suicide, a preoccupation with thoughts of hurting another person and a clear plan as to how they would follow through on those thoughts. The individual demonstrates poor judgment and does not provide evidence of protective factors.

Individuals who are assessed as high risk for harming others will be encouraged to access supportive health services, e.g. calling Lifeline (13 11 14) or other external support services in their local area, or their current mental health professional, if they are already engaged with a service. In some cases of moderate to high risk, the participant may have their access to Peer Tree temporarily suspended.

Depending on the immediacy of the risk, research members may decide to access health and emergency services and/or an emergency contact on the individual’s behalf. This will only be done with their permission, unless in the highly unlikely scenario arises that the person is unwilling for services to be contacted and the person presents a high risk to themselves or others. In this circumstance, the research will have a duty of care to contact services without the person’s permission. This possibility is highlighted in the participant information sheet when the participant completes the baseline assessment.

The researchers are advised to seek supervision from a senior research member (Dr Michelle Lim) to discuss participant’s wellbeing, the nature of the risk, adherence to protocol and outcome of the situation.

## iii) Risk of harm or damage to objects or property

Individuals who report any level of risk to harm or damage to objects or property will not be offered access to Peer Tree at the baseline assessment. However, the nature of risk could vary throughout their participation in the research program. If risk of harm or damage to objects or property occurs during a participant’s involvement in the study, the clinical moderator and principal investigator should be notified. The clinical moderator will talk to the participant over the phone to assess the current level of risk to objects or property and encourage the participant to access supportive health services, e.g. calling Lifeline (13 11 14) or other external support services in their local area, or their current mental health professional, if they are already engaged with a service.

Depending on the immediacy of the risk, research members may decide to access health and emergency services and/or an emergency contact on the individual’s behalf. This will only be done with their permission, unless in the highly unlikely scenario arises that the person is unwilling for services to be contacted and the person presents a high risk to themselves or others. In this circumstance, the research will have a duty of care to contact services without the person’s permission. This possibility is highlighted in the participant information sheet when the participant completes the baseline assessment.

The researchers are advised to seek supervision from a senior research member (Dr Michelle Lim) to discuss participant’s wellbeing, the nature of the risk, adherence to protocol and outcome of the situation.

## Additional Risk Assessment Protocol:

These questions may help gather relevant information to inform the assessment, however it is important to listen to the participant and ask relevant and appropriate questions. Possible questions to consider in completing a risk assessment related to suicidal thoughts or risk to others or objects:

- What are the thoughts?
- How often do the thoughts occur?
- How long do the thoughts last?
- Can the person control the thoughts when they arise?
- What has happened since these thoughts commenced?
- How likely is the person to follow through with the thoughts?
- What might have stopped the person from acting on the thoughts?
- Has the person thought about a plan to end their life or hurt others? (If yes, what, when, how, where. Is it carefully planned or impulsive?)
- Does the person have access to the means to follow through with the plan?
- How likely is the person to follow through with the plan?
- What might have stopped the person from acting on the plan?
- Have they told anyone? Who have they spoken to?
- Are there any supports able, available and acceptable now to the person?
- Have they sought any treatment or support from a medical or mental health service? Who, when, where. Are they still engaged with the service? Is it helpful? Is it something the person may do again in the future, if required?
- Are there any strengths or coping strategies that can be used to help keep the person safe/manage the thoughts?
- Does the person have a safety plan? Can the person verbalise the safety plan such as helpful coping strategies, contact persons, reasons for living etc.?

Please refer to the risk assessment section above for the steps to take in regard to the level of risk identified.

## iv) Risk of mental health deterioration

Participants currently experiencing mental ill-health will not be eligible to participate in the Peer tree program at the time of the baseline assessment. However, it is important the clinical moderator continues to monitor and be aware of any potential changes in participant’s mental health and well-being.

A moderator may become aware of a change in a participant’s mental health and well-being through:

- The participant explicitly stating concerns, and/or;
- Observing or noticing through the chat forum that the participant is responding differently and may suggest he or she is unwell.

If another moderator becomes aware of any participant being at risk of mental health deterioration, the moderator is required to immediately notify the clinical moderator and principal investigator to assess the concerns.

If this situation arises, the clinical moderator will contact the participant via a phone call to check in with how the participant is going in regard to their mental health and wellbeing. The clinical moderator will support the participant to access mental health services such as calling Lifeline (13 11 14), Swinburne Psychology Clinic, other external support services in their local area, or their current mental health professional, if they are already engaged with a service.

If the clinical moderator has uncertainty about the participant’s mental health and well-being and the necessary steps to undertake, or the clinical moderator is concerned about the impact of the Peer Tree program on their mental health and well-being, the clinical moderator should seek immediate supervision from the senior staff. The first point of contact will be Dr Lim, followed by Dr Eres.

Depending on the immediacy of the deterioration, research members may decide to access health and emergency services and/or an emergency contact on the individual’s behalf. This will only be done with their permission, unless in the highly unlikely scenario arises that the person is unwilling for services to be contacted and the person presents as unwell and a potential high risk to themselves or others. In this circumstance, the researcher will have a duty of care to contact services without the person’s permission. This possibility is highlighted in the participant information sheet when the participant completes the baseline assessment.

The researchers are advised to seek supervision from a senior research member (Dr Michelle Lim) to discuss participant’s wellbeing, the nature of the risk, adherence to protocol and outcome of the situation.

## v) Participant admitted to hospital.

If the moderators become aware of a participant being admitted to hospital during their participation in the program, the research or clinical moderator will give the participant a phone call to check in and find out additional information about their admission such as the context of the admission, length of stay, current mental and physical wellbeing. This information will help the moderators develop an understanding of the participant’s current situation and talk with the participant about their involvement in the study. The moderator will ask the participant whether he/she would like to continue in the program or would like to take a break to focus on their health. Participants will have the option to make the decision, however, in the event the moderator is concerned about the impact of their involvement of the study on the participant’s wellbeing, the moderator should talk with Dr Lim (or Dr Eres) and seek supervision to determine how best to support the participant and ensure their involvement in the study will be beneficial for them and not lead to any negative impact.

Participants who temporarily pause their involvement in the program (e.g., whilst in hospital) may return to engaging with Peer Tree when they are ready. The moderator will record the date at which the participant stopped using the program and when the participant re-commenced using the program for the study records.
